# The Riffomonas YouTube Channel: An Educational Resource To Foster Reproducible Research Practices

**DOI:** 10.1128/mra.01310-22

**Published:** 2023-01-18

**Authors:** Patrick D. Schloss

**Affiliations:** a Department of Microbiology and Immunology, University of Michigan, Ann Arbor, Michigan, USA; Indiana University, Bloomington

## Abstract

Methods for analyzing data in a reproducible manner are often viewed as impenetrable to scientists more familiar with laboratory research. The Riffomonas YouTube channel is committed to teaching these scientists and others how to engage in reproducible research using modern data science tools.

## ANNOUNCEMENT

As high-throughput data generation becomes more common in microbiology and other disciplines, there is a significant need for laboratory scientists to develop data science skills (1). Unfortunately, traditional undergraduate and graduate biology training programs are often deficient in opportunities for scientists to develop the skills necessary to analyze large datasets in a reproducible and robust manner (2, 3). Numerous organizations seek to fill this void, including the Carpentries, Codecademy, and DataCamp (4). There are also numerous video tutorials available on YouTube. Although the content available through these platforms is popular, there has been a gap in content that emphasizes project-based learning.

The Riffomonas YouTube channel (https://www.youtube.com/c/RiffomonasProject) seeks to fill this gap. I started consistently posting videos at the beginning of the coronavirus disease 2019 (COVID-19) pandemic in April 2020. As of the end of November 2022, the channel had 11,327 subscribers and included 285 videos that had been viewed 635,947 times. The majority of these are 264 videos in the “Code Club” playlist (5) (Table 1). Other videos are related to a previously described tutorial series on reproducible research (6) and series in which reproducible research practices are used to address topical questions. Code Club videos are typically between 20 and 30 min long. The code that is developed in the videos is available through a website (https://riffomonas.org/code_club/) and the channel’s GitHub-hosted account (https://github.com/riffomonas).

**TABLE 1:** Playlists found on the Riffomonas YouTube channel^*a*^

Topic	Playlist title	No. of videos
Data science	Data visualization with R’s tidyverse and allied packages	146
	Data manipulation within R’s tidyverse and other packages	116
	Data analysis with base R	39
	Tools for reproducible data analysis	33
	Working at the command line	26
	Literate programming with R markdown	18
	Machine learning with mikropml R package	16
	Version control with Git and GitHub	15
	Scientific writing	15
	Project organization	3
Project-based series	All Code Club videos since 2 April 2020	265
	Microbiome data analysis and visualization	86
	ASV/OTU^*b*^ sensitivity and specificity analyses	67
	Visualizing COVID-19 vaccination attitudes	31
	Climate change data visualization	29
	Evaluating rarefaction and its alternatives	18
	Drought index visualization	17
	Reproducible research tutorial series	14
	Commemorating Juneteenth 2022 with a visualization	5
	2018 MLB All Star Break data analysis sprint	4

a Because most videos cover more than one topic, they are found in multiple playlists. Playlists and counts were current as of 1 December 2022. Playlists can be found under the Playlists tab at https://www.youtube.com/c/RiffomonasProject.

b ASV, amplicon sequence variant; OTU, operational taxonomic unit.

The channel name, Riffomonas, comes from the concept of riffing, in which musical themes are adapted to achieve a similar sound, albeit perhaps in different contexts (6). This is to emphasize the value of reproducibility not only for recreating a set of results but for applying a method with a different data set (7). The channel covers topics related to reproducible data analysis practices, including R programming, data visualization, project organization, version control, command line programming, workflow tools, and scientific publishing (Table 1). Each video includes a brief introduction followed by me live coding to achieve a goal. I emphasize the use of live coding to modulate the rate of instruction and to show viewers my own coding practices. Observing an experienced analyst make mistakes normalizes some level of failure and demonstrates the strategies they can use to resolve their own mistakes. Viewers are encouraged to follow along with each video and to apply the new information to their own project.

Each video emphasizes a specific topic but includes other content that is selected to review topics covered in recent videos. Although videos can be watched individually, they often form a project arc (Table 1). For example, between July 2020 and July 2021, I formulated a research question, obtained and analyzed data to answer the question, and wrote a paper that was published in *mSphere* (8). This series of 67 videos covered every topic from creating the initial directory on my computer to house the project files through reviewing the proofs of the published manuscript. Other project arcs have included visualizing microbiome data, modeling microbiome data using machine learning tools, analyzing the impacts of rarefying microbiome data, and other topics. Going forward, the Riffomonas channel will continue to post project-based content to help researchers develop their reproducible research skills.

### Data availability.

The Riffomonas YouTube channel is available at https://www.youtube.com/c/RiffomonasProject. The code developed in the Code Club videos is available at https://riffomonas.org/code_club/ and the channel’s GitHub-hosted account (https://github.com/riffomonas).
